# Molecular Characterization and Cluster Analysis of SARS-CoV-2 Viral Isolates in Kahramanmaraş City, Turkey: The Delta VOC Wave within One Month

**DOI:** 10.3390/v15030802

**Published:** 2023-03-21

**Authors:** Nadia Marascio, Merve Cilburunoglu, Elif Gulsum Torun, Federica Centofanti, Elida Mataj, Michele Equestre, Roberto Bruni, Angela Quirino, Giovanni Matera, Anna Rita Ciccaglione, Kezban Tulay Yalcinkaya

**Affiliations:** 1 Department of Health Sciences, Institute of Microbiology, “Magna Grecia” University, 88100 Catanzaro, Italy; 2 Microbiology Department, Faculty of Medicine, Kahramanmaras Sutcü Imam University, 46050 Kahramanmaras, Turkey; 3 Department of Applied Clinical Sciences and Biotechnology, University of Aquila, 67100 L’Aquila, Italy; 4 Instituti i Shendetit Publik (ISHP), 1000 Tirana, Albania; 5 Department of Neurosciences, Istituto Superiore di Sanità, 00161 Rome, Italy; 6 Department of Infectious Diseases, Istituto Superiore di Sanità, 00161 Rome, Italy

**Keywords:** COVID-19, SARS-CoV-2, delta VOC, unusual mutations, cluster identification

## Abstract

The SARS-CoV-2 pandemic has seriously affected the population in Turkey. Since the beginning, phylogenetic analysis has been necessary to monitor public health measures against COVID-19 disease. In any case, the analysis of spike (S) and nucleocapsid (N) gene mutations was crucial in determining their potential impact on viral spread. We screened S and N regions to detect usual and unusual substitutions, whilst also investigating the clusters among a patient cohort resident in Kahramanmaraş city, in a restricted time span. Sequences were obtained by Sanger methods and genotyped by the PANGO Lineage tool. Amino acid substitutions were annotated comparing newly generated sequences to the NC_045512.2 reference sequence. Clusters were defined using phylogenetic analysis with a 70% cut-off. All sequences were classified as Delta. Eight isolates carried unusual mutations on the S protein, some of them located in the S2 key domain. One isolate displayed the unusual L139S on the N protein, while few isolates carried the T24I and A359S N substitutions able to destabilize the protein. Phylogeny identified nine monophyletic clusters. This study provided additional information about SARS-CoV-2 epidemiology in Turkey, suggesting local transmission of infection in the city by several transmission routes, and highlighting the necessity to improve the power of sequencing worldwide.

## 1. Introduction

Coronavirus Disease-19 (COVID-19), caused by the Severe Acute Respiratory Syndrome Virus 2 (SARS-CoV-2), is characterized by clinical manifestations ranging from asymptomatic and mild symptoms to severe disease and death [1]. The time from virus exposure to the host response and development of symptoms is called the window period, and clinical manifestations may appear 2 to 14 days after the viral attack [1]. Potential complications, leading to worse outcome, include pneumonia, respiratory failure, and cardiovascular and neurological diseases, which, in addition to the use of antiviral drugs, require supportive care such as oxygen and mechanical ventilation [1,2]. The SARS-CoV-2 pandemic has seriously affected the population in Turkey, a country acting as a bridge between Asia and Europe [3]. The country adopted public health measures before the first case detected on March 11th, 2020, and before the pandemic was declared by the World Health Organization (WHO). In order to the arrest spread of infection, a curfew was initially imposed on people over 65 years old, and subsequently to subjects with chronic diseases and those under 20 years old [4,5]. Since April 2020, several public health interventions have been implemented, such as restrictions on entering or leaving the city and scientific and cultural meetings, or national and international activities were postponed. Social distancing was introduced for restaurants (only takeaway service), whilst barber shops and beauty centers were suspended and sporting activities were held without the public [5]. In early 2021, the first vaccine was approved for “urgent use”, and within 23 days, the vaccinated population was 3.04%. In this regard, the vaccination campaign was conducted in four steps, giving priority to fragile subjects and healthcare workers [6]. In June 2021, the capacity to diagnose SARS-CoV-2 infection by real-time reverse transcription–polymerase chain reaction (RT-PCR) was expanded to all 81 provinces, increasing the number of laboratories able to perform molecular tests to 482 [5]. Real-time RT-PCR carried out by nasopharyngeal/oropharyngeal swab is considered the gold standard to detect SARS-CoV-2 RNA and to provide quantitative information using the cycle threshold (Ct) values of the viral load [7]. 

Alongside timely diagnosis, the WHO and European Centre for Disease Prevention and Control (ECDC) advised to detect variant circulation using SARS-CoV-2 whole-genome or spike (S), partial or complete, gene sequencing [8]. Phylogenetic analysis of newly generated sequences combined with epidemiological and clinical data was a key tool to understand the transmissibility pathway of this pandemic virus and to manage public health measures against COVID-19 disease [9,10]. In March 2020, the Global Initiative on Sharing All Influenza Data (GISAID) database started to share SARS-CoV-2 genomes from Turkey, contributing to the performance of molecular characterization and to evaluate the prevalence and diffusion of variants in this specific geographic area [4]. During the pandemic waves, several SARS-CoV-2 variants were identified and subsequently classified as variants of concern (VOC), variants of interest (VOI), and variants under monitoring (VUM) by the WHO [8]. In May 2021, the prevalent variant circulating in Turkey was Alpha (B.1.1.7), followed by the Beta (B.1.351) and Gamma (P.1) VOCs and, by a low percentage, of the Epsilon (B.1.427 and B.1.429) VOI [4]. In June 2021, COVID-19 cases increased rapidly (around four million subjects infected) and over 40,000 deaths were observed [4]. The hallmark of the fourth wave was the appearance of the Delta (B.1.617.2) lineages [11]. Compared to other VOCs, Delta is highly transmissible and also responsible for more severe disease. Additionally, this VOC increased the hospitalizations and death of infected subjects, in particular in the older age group, regardless of vaccination status [12,13]. In August 2021, more than 20,000 people were infected per day in Turkey [12]. Delta dominated the epidemic worldwide until Omicron emerged in November 2021. At present, Omicron, including its sublineage and recombinant forms, has a decreased disease severity and infection cases are characterized by sore throat due to lower viral replication in the gut and lung cells [14].

Molecular characterization has allowed the identification of genomic substitutions responsible for increased transmissibility and pathogenicity of the SARS-CoV-2 enveloped virus [4,14]. The SARS-CoV-2 single-stranded RNA (ssRNA), positive-sense genome (around 30 kb), is structured from 5′ to 3′ as follows: the untranslated region, open reading frame 1ab (ORF1ab), spike (S), ORF3ab, envelope (E), membrane (M), ORF6, ORF7ab, ORF8, nucleocapsid (N), ORF10, and untranslated region. The ORFs encode for structural and nonstructural proteins [15]. The transmembrane glycoprotein S, necessary for entry into the host cells, contains two subunits, S1 and S2, able to engage the host cell receptor, angiotensin-converting enzyme 2 (ACE2) by the receptor-binding domain (RBD), and to mediate fusion with host cell membranes, respectively [15]. RBD is the main target of neutralizing antibodies and viral RNA is under constant mutation to adapt to the host immune system [14]. SARS-CoV-2 encodes an exonuclease that repairs genome error during viral replication and the mutation rate per site per year is between 1.12 and 6.25 × 10^−3^ [13]. Interestingly, viral isolates from Turkey showed the third-highest number of mutations in the S gene and more mutations in the ORF1ab gene compared to other reference genomes available in public databases [16]. The SARS-CoV-2 genome showed a high-mutational rate [16]. The analysis of mutations in the S gene is crucial to determine their potential impact on the structure and function of the protein and to monitor potentially emerging variants that might be responsible for greater virus transmissibility and reinfection rates, as well as vaccine or treatment failure [16,17,18]. On the other hand, recent data highlight the importance of the N protein, which interacts with the viral M protein to promote the anchoring of ribonucleoprotein particles and the recognition of viral RNA [19,20,21]. The variability of the N protein is relatively high, with more than 86% of amino acids that can be substituted when considering SARS-CoV-2 genetic diversity [21]. Recently, the atypical N G214-G215 deletion identified on the Delta VOC led to the failure of SARS-CoV-2 diagnosis by commercial molecular assays [22]. Sahin and coworkers, analyzing sequences from Turkey, detected 21 mutations in the N regions and no deletions [14]. Alteration of the three-dimensional structure of the N protein could impact on pathogenesis, improving viral transcription, assembly, and infectivity [19,21]. In this view, the N protein could be considered as a target for vaccine development [14]. 

This study was designed to focus on a selected patient cohort resident in Kahramanmaraş city in August 2021. The city is located in southern Turkey and is the capital of Kahramanmaraş Province. The population living in the metropolitan area was 560,000 in 2021 and 583,000 at present. During routine diagnosis by molecular tests, the S region was sequenced, as suggested by the WHO, to identify new SARS-CoV-2 circulating variants, while the N region was analyzed as an important genomic hotspot. Herein, we screened the spike and nucleocapsid whole regions by Sanger sequencing to detect usual and unusual SARS-CoV-2 mutations carried on newly generated sequences. Additionally, we investigated the clusters occurring over a one-month period in the cohort by phylogenetic analysis using concatenate S and N genomic regions. 

## 2. Materials and Methods

### 2.1. Sample Collection and Diagnostic RT-PCR 

Fifty-eight nasopharyngeal swab samples (collected between 3rd and 24th August 2021) from subjects attending to the Kahramanmaraş Sütçü İmam University Health Practice and Research Hospital Microbiology Laboratory, Turkey, with available clinical data, were included in the analysis. All patients with COVID-19 prediagnosis were confirmed to be SARS-CoV-2-RNA-positive. Viral RNA was detected by a real-time RT-PCR assay, targeting the ORF1ab, nucleocapsid (N), and spike (S) genes (Bio-Speedy SARS-CoV-2 Emerging Plus, Istanbul, Turkey). The analytical sensitivity of the Bio-Speedy kit reported by the manufacturer is 98%. The Ct value is inversely related to the viral RNA copy number in clinical samples. Values of 12 ≤ Ct < 20, 21 ≤ Ct ≤ 25, and Ct > 25 were associated with high, medium, and low viral load, respectively. 

### 2.2. Sanger Sequencing

Viral RNA isolation was performed with the HibriGen General RNA Isolation Kit (Kocaeli, Turkey). The purified RNA was reverse-transcribed into complementary DNA (cDNA) using the OneScript Plus cDNA Synthesis Kit (Applied Biological Materials, Richmond, BC, Canada). PCR was performed in a 20 μL reaction mix, as follows: 2 mM MgCl_2_, 0.2 mM dNTPs, 0.2 µM forward and reverse primers, and 1U Taq DNA polymerase Solis HOT FIREPol (Solis Biodyne, Tartu, Estonia). The S and N genes were amplified and sequenced using primers previously reported [23]. Sanger sequencing was performed using Genetic Analyzer 3130 XL (Applied Biosystems, Carlsbad, CA, USA) and with the BigDye Terminator v3.1 Cycle Sequencing kit (Life Technologies Corporation, Austin, TX, USA) according to manufacturer’s protocol. Consensus sequences by combining electropherograms were generated in the Sequencher 5.4.6 software (Gene Codes Corporation, Ann Arbor, MI, USA). 

### 2.3. Viral Classification and Mutational Analysis

SARS-CoV-2 viral isolates were classified by Pangolin COVID-19 Lineage Assigner v.4.0.6 [24]. Amino acid changes of spike and nucleocapsid sequences were analyzed using the CoVsurver and Stanford Coronavirus Resistance Database online tools [25,26]. The amino acid changes were further confirmed comparing newly identified isolates to the SARS-CoV-2 Wuhan-Hu-1 reference sequence (accession NC_045512, version NC_045512.2) downloaded from the GenBank^®^ database [27]. 

### 2.4. Phylogenetic and Cluster Analyses

SARS-CoV-2 genomes of Turkish strains, submitted between 1st and 31st August 2021, were downloaded from the GISAID database. High-coverage Delta (B.1.617.2) VOC sequences without nucleotide gaps and with complete collection dates were included in the analysis. Duplicate sequences identified by the ElimDupes tool were excluded [28]. The reference sequences from the whole country included in the analysis had all been submitted by the Turkish Ministry of Health. To perform the molecular analysis, the 58 newly generated S (3821 nucleotides) and N (1259 nucleotides) sequences were concatenated to increase the overall nucleotide positions being compared between isolates and, therefore, to increase the chance of potential differences that could allow for better classification. A total of 99 representative reference sequences were aligned to the 58 concatenated sequences and to NC_045512.2 (included as outgroup) by MAFFT [29]. The complete dataset, including 158 sequences (final length 5080 nucleotides), was edited by MEGAv7 [30]. The maximum likelihood (ML) tree was estimated using a GTR + G nucleotide substitution model by the IQtree software, with 1000 bootstrap replicates and a 70% cut-off definition to detect clusters [31]. The phylogenetic tree was visualized using FigTree v1.4.2 [31].

## 3. Results

### 3.1. Patient Characteristics and ROUTINE Diagnosis

All patients were positive for both genomic regions by the real-time RT-PCR assay. The median Ct value of 14.96 (ranging from 10.62 to 21.21) was detected for the ORF1ab plus N genomic regions, while a median Ct value of 17.35 (ranging from 13.02 to 23.34) was detected for the S region. Overall, the median Ct value for both regions was less than 25 (ranging from 10.62 to 23.34), identifying a high viral load in all nasopharyngeal swabs. The median age of the 58 patients was 33.9 years (range 0–78) and the percentage of females was 53.4%. Specific COVID-19 symptoms were referred by most of the patients (58.6%). Unfortunately, vaccination status was not available. Demographic and clinical characteristics of the patients at diagnosis are reported in Table 1. 

### 3.2. Classification and Mutation Pattern of Isolates

**Figure 1:**
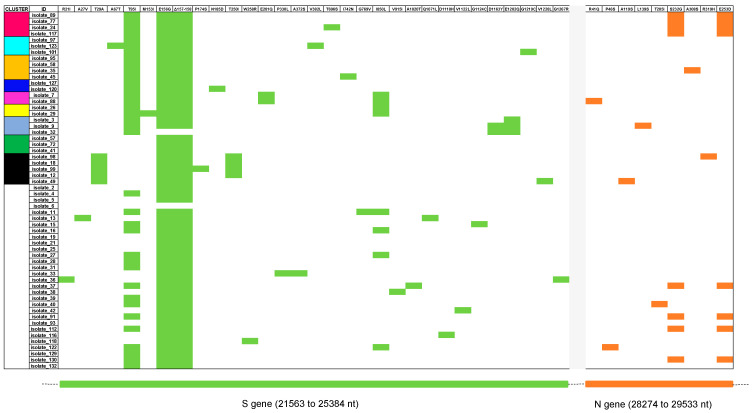
Heatmap of S (light green) and N (orange) amino acid substitutions detected in each isolate. The appearance of substitutions was organized based on their inclusion in a cluster (colored box) or not (white box) by phylogenetic analysis. Nucleotide (nt) position of sequenced genes is reported in the lower part of the figure.

The 58 consensus sequences were all classified as Delta VOC, B.1.617.2 lineage, by the PANGO Lineage tool. Any B.1.617.2 genome was classified as a unique AY sublineage by a genotyping online tool. The S and N regions of the newly identified isolates showed several amino acid mutations. All isolates carried the T19R, G142D (with only one exception coding G), L452R, T478K, D614G, P681R, and D950N spike amino acid substitutions. Notably, some isolates carried one or more unusual mutations, such as A67T, P330L, A372S, T696S, I742N, V915I, A1020T, G1124C, and G1267R. Thirty-six out of fifty-eight (62.1%) isolates showed the T95I mutation. The N proteins of the 58 isolates displayed the usual D63G, R203M, G215C, and D377Y amino acid substitutions, and in one isolate, the L139S unusual mutation. The specific mutational pattern of whole S and N genomic regions for each isolate is reported in Figure 1.

To check the prevalence of the unusual L139S mutation, 52 more N sequences of the Delta VOC collected during the same time span were screened, and again only one isolate carried this amino acid substitution. Additionally, we identified two T24I, one A359S, and one P364L N mutations among the fifty-two isolates. 

### 3.3. Cluster Investigation

**Figure 2:**
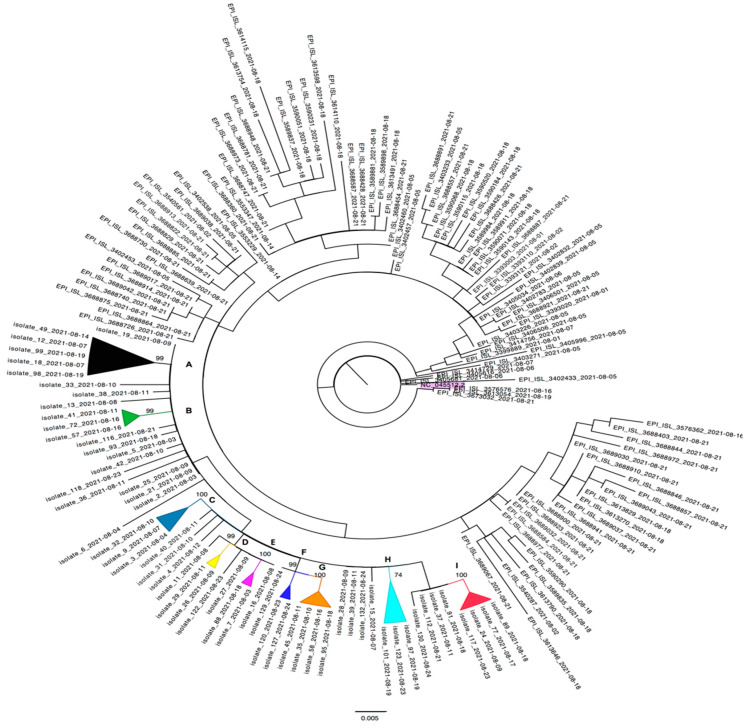
ML phylogenetic tree was estimated using 99 sequences from Turkey and 58 SARS-CoV-2 newly generated sequences. The tree was rooted by the midpoint rooting according to the NC_045512.2 (highlighted in purple) outgroup sequence. The reliability of the phylogenetic clustering was evaluated using bootstrap analysis with 1000 replicates. Bootstrap support values (>70%) are only shown for the clusters (colored collapsed cartoons) including Kahramanmaraş city isolates. The nine clusters were identified with the alphabetic letters from A to I. The scale bar at the bottom of the figure represents genetic distance (0.005).

The ML phylogenetic tree of the final dataset showed the presence of nine statistically significant monophyletic clusters, including only sequences from Kahramanmaraş city. No clusters among the new isolates and sequences from the rest of Turkey were observed (Figure 2).

The nine clusters (from A to I) contained between two to five patients, with a median cluster size of three positive subjects. Thirty out of fifty-eight (51.7%) new isolates did not belong to any cluster, including the two isolates detected in a married couple (#19 and #25 isolates).

Cluster A (black) comprised 5/58 isolates, all of them carried the T29A, E156G, and Del157-158 S mutations, while 4/5 carried the T250I, 1/5 (isolate #99) carried the P174S, and 1/5 (isolate #49) carried the V1228L S mutations. The A119S and R319H N substitutions were reported in isolate #49 and isolate #98, respectively. The five sequences belonged to two males and three females, who reported COVID-19 symptoms in 3/5 cases. Cluster B (green) included three (#41, #57 and #72) isolates, displaying the E156G and Del157-158 S mutations, from patients (two males and one female) who reported COVID-19 symptoms. Cluster C (turquoise) showed very high bootstrap support (>100) and comprised three isolates carrying the T95I and E1202Q S mutations. In particular, isolate #3 displayed the S E156G and Del157-158 substitutions, while isolate #9 also carried the D1163Y S plus unusual L139S N mutations. The isolate #32 carried the D1163Y S substitution as well. Only the patient infected by isolate #9 reported COVID-19 symptoms, while the patient infected by isolate #3 reported an atypical backache. Cluster D (yellow) included two isolates carrying the T95I, E156G, Del157-158 and I850L S substitutions. Isolate #29 was also characterized by the M153I S mutation. Both isolates belonged to patients with specific symptoms at diagnosis.

From Cluster E to Cluster I, all isolates carried the T95I, E156G, and Del157-158 S substitutions. Isolates #7 and #88 of Cluster E (fuchsia) additionally showed E281Q and I850L in the spike region, while isolate #88 also showed the R41Q N mutation. Isolate #120, but not the isolate #127, of Cluster F (dark blue) displayed the N185D S mutation. Inside Cluster G (orange), only isolate #45 was characterized by the I742N S substitution. The isolates #101 and #123, forming Cluster H (light blue) with isolate #97, carried the peculiar G1219C and A67T plus V382L S substitutions, respectively. 

Cluster I (red) is characterized by S232G and E253D N mutations. In particular, isolate #24 also carried the T696S spike amino acidic substitution. All four patients infected by isolates included in this cluster reported specific COVID-19 symptoms.

Despite the highest prevalence of infection being in the 10–19 (14/58) and 30–39 (14/58) age groups, clusters were not significantly associated with age. The prevalence of infection was similar between males and females (46.6% vs 53.4%) in our cohort and in each cluster.

## 4. Discussion

This study aimed to evaluate the genetic variability amongst 58 newly generated sequences collected during routine diagnosis within a patient cohort resident in Kahramanmaraş city in August 2021. In addition, cluster analysis among viral strains isolated from patients was performed in a restricted time span (one month) to understand the dynamics of SARS-CoV-2 transmission during the fourth wave in Turkey. At the time of our analysis, the pandemic was characterized worldwide by the Delta VOC [8]. The SARS-CoV-2 infectivity increased by 60–70% compared to Alpha, and the incubation period decreased, ranging from 4.4 to 5.8 days. This VOC appeared to cause more hospitalizations (60%) and deaths (around 3%) than previous variants and escaped neutralizing or polyclonal antibodies elicited by previous immunity (natural infection or vaccination) [13]. Delta was responsible for breakthrough infections in 28% of cases, and among them, fully vaccinated subjects had similar viral loads to nonvaccinated people [32]. It is noteworthy that, the increase in Delta viral infectivity and replication produced more RNA copies/milliliter than non-Delta variants [13].

In our cohort, diagnosis was performed using the gold standard method, RT-PCR, despite several methods that were developed to detect SARS-CoV-2 RNA in respiratory and saliva specimens [7]. To diagnose COVID-19 infections, the U.S. Food and Drug Administration (FDA) authorized many commercial tests, each of which has its own accuracy (specificity and sensitivity), usually stated by manufacturers [7,33]. We used the Bio-Speedy kit with a 98% sensitivity, as its performance agrees with other molecular assays, such as the Panther Fusion SARS-CoV-2 with a 98.3% accuracy [33]. Furthermore, a community-based testing study compared the Panther Fusion to the Lucira test, achieving a 98% negative percent agreement, using nasopharyngeal swabs [33]. The 58 patients were positive with Ct values <25 (medium viral load) for the screened ORF1ab/N and S genes, in particular the Ct value of ORF1ab/N target was <21, while the Ct value of the S target was <23. The Ct value was not, however, significantly related to COVID-19 symptoms at the time of diagnosis, as previously reported by Guney and coworkers [34]. Different studies showed that the ORF1ab (median 7.83 log10 copies/mL) and N (median 7.69 log10 copies/mL) Delta targets had higher viral loads than historical variants. The median Ct value for Delta infections was 23, significantly lower than wild-type N genes (Ct 36) [13]. On the other hand, the performance of antigenic assays is lower compared to the gold standard [7]. The Ellume antigenic test, performed on nasal swab, and the Innova rapid lateral flow COVID-19 test, carried out on nasal or throat samples, correctly identified 96% of individuals with COVID-19 symptoms and 95% of participants with high viral loads, respectively [33]. Finally, in a study conducted on 251 hospitalized adults, the FebriDx system, a finger-prick blood test, showed 93% sensitivity and 86% specificity [33].

The genetic variability of SARS-CoV-2 determined the accumulation of mutations over time, influencing transmission, severity of disease, and vaccination efficacy [17,35]. The genomic evolution of variants could be associated with the plasticity of viral proteins related to ssRNA^+^ position [21]. Since the beginning of the pandemic, clinical and demographic characteristics linked to sequence information were noted to better understand outbreak episodes and local dynamic evolution [36,37]. With this in mind, we sequenced the S and N regions of autochthonous viral strains. All cDNA samples were successfully amplified using endpoint PCR and sequenced by Sanger methods. All viral isolates were identified as the Delta VOC (B.1.617.2 lineage) by the PANGO Lineage classification tool. Phylogenetic analysis using the concatenated sequences of highly variable S and relatively high-variable N regions identified nine statistically supported (bootstrap >70%) clusters, including from two to five patients. In particular, two isolates, #19 and #25 from a couple, were intermixed with sequences from the metropolitan area, highlighting a different transmission event. However, each cluster showed a pattern of nucleotide mutations and a unique signature close to the well-known amino acidic substitutions characterizing the Delta VOC [8]. The clusters did not show transmission among specific age groups, suggesting several routes of transmission. Furthermore, the thirty patients not included in any specific cluster were part of the metropolitan area clade not intermixed with other sequences coming from out-of-town. The same high percentage (24.1%) of infections was found among the 10–19 and 30–39 age groups, and 58.6% (34/58) of patients reported specific COVID-19 symptoms. In our cohort, infected adults and young adults showed similar clinical manifestation. According to literature data, the Delta variant affected a higher proportion of younger adults (under 20 years) in comparison to the wild-type strain and older age was related to severe cases [38]. 

The S protein of all new isolates carried the T19R, G142D (only one exception with G), L452R, T478K, D614G, P681R, and D950N usual substitutions of the Delta VOC. Interestingly, just one isolate did not display the G142D mutation, which interferes with the neutralization of many N-terminal-domain (NTD)-binding monoclonal antibodies (mAbs) [26]. The L452R amino acid change was maintained by the Omicron BA.4 and Omicron BA.5 VOC [39]. This mutation is associated with a reduction in susceptibility to several anti-SARS-CoV-2 mAb treatments [39]. The T478K, also a typical mutation of the Omicron VOC, is an RBD mutation apparently without effect on mAbs or convalescent plasma vaccines. The D614G increased in prevalence in late February 2020 and is currently the most prevalent form worldwide [40]. The virus carrying the D614G mutation was responsible for increasing the number of S proteins per each virion [14,40]. The P681R mutation was associated with an increased cleavage rate of S1/S2 subunits, the main sites responsible for the infection of the host cells, resulting in better transmissibility [14,41]. Viral isolates from Turkey are characterized by frequent mutation loads and proteins have undergone several mutations since the outbreak of the COVID-19 pandemic [39]. Interestingly, 36/58 (62.1%) isolates carried the T95I S mutation, reported with low prevalence worldwide in the Delta VOC, but associated with a significant increase in viral fitness [41,42].

Furthermore, nine unusual mutations were detected from the S protein and one from the N protein. One patient (isolate #24) carried the T696S mutation in the S1/S2 cleavage site. This polybasic cleavage site is strictly related to the ability of SARS-CoV-2 to fuse with cell membranes and to start the infection process [40]. Another mutation found within the spike region was A67T (isolate #123), associated with the Eta (B.1.525) VOI according to the WHO classification based on amino acid substitutions observed in 303,250 spike-protein sequences [39]. The G1267R (isolate #36) and V915I (isolate #38) substitutions were previously seen in SARS-CoV-2 spike-protein sequences analyzed from Indian isolates in different domains, cytoplasmic regions, and Heptad repeat region 1 (HR1), respectively [43]. The HR1, contained in the S2 domain, is usually more conserved than other regions of the S protein and is a candidate to inhibit virus fusion [44]. The S protein displayed additional mutations, including G1124C, located in β hairpin, (isolate #15), P330L, located in RBD, and A372S (isolate #33), A1020T, located in central helix, (isolate #37), and I742N (isolate #45), which were reported worldwide with a prevalence below 0.01% [26]. Noteworthy, the majority of unusual P330L, A372S, I742N, V915I, A1020T, G1124C, and G1267R substitutions were displayed in sequences not included in the nine clusters, suggesting a random selection in single isolates. Further studies are required to evaluate the impact on protein conformation and the stability of carrying these mutations, the location in a relevant site, and their possible spread.

All 58 new isolates displayed in the N protein the four (D63G, R203M, G215C, D377Y) amino acid substitutions peculiar to the Delta VOC [25]. In particular, the G215C mutant was identified as responsible for secondary-structure alteration [21]. The atypical N G214-G215 deletion was not identified in any new isolates [22]. The isolate #9 carried the unusual L139S mutation. Given the role of the SARS-CoV-2 N protein in the transcription of viral RNA, viral replication, and virion assembly, we screened 52 more N sequences for the presence of the L139S substitution, which was found in only one more isolate analyzed [4,45]. This observation suggests that the L139S substitution may be considered a neutral variant that emerged in patients residing in Kahramanmaraş city that did not spread. Few isolates showed the T24I (2/52) and A359S (1/52) mutations, which were predicted to destabilize the N protein structure. On the contrary, the P364L N mutation, found in one isolate, was reported as a substitution stabilizing the nucleocapsid structure [19]. However, the detected N mutations did not affect the real-time RT-PCR efficiency, providing false-negative results, as previously reported by Nalla and colleagues, who analyzed lower PCR sensitivity in different countries [46]. The molecular assay performance was not influenced by the mismatch between the autochthonous sequences and primer–probe used in clinical practice [20]. In a previous study, the typical R203M position in the nucleocapsid protein was found mutated as R203K in twenty Turkish sequences due to two consecutive nucleotide changes. The 204 position was also mutated (G204L) [4]. Interestingly, Cluster I was characterized by S232G plus E253D N substitutions not yet reported in the literature data. This cluster was supported by 100% of bootstrap and probably it was related to a single event of infection. The N gene mutational rate is high; Rahman and colleagues reported 46.07% of amino acid substitutions after analyzing 67,124 (complete or near-complete) sequences in 145 different countries retrieved from GISAID. Overall, it is important to continuously monitor its ongoing evolution for diagnostic intervention and therapeutic approaches. The N protein as a vaccine target is a challenge [20]. 

SARS-CoV-2 phylogenetic and mutational analyses were, and still are, of paramount importance to slow the diffusion of VOCs in the age of globalization. In Indonesia, Delta was the dominant variant in the second wave (May–August 2021), carrying unique substitutions that differ from those of the first wave [47]. The increased transmissibility in the second wave was related to key genomic mutations, such as T19R, T95I, G142D, L452R, T478K, D614G, P681R, and D950N (also found in the S protein of our isolates) held responsible for massive outbreaks worldwide [47]. During the summer of 2021, in Ukraine, molecular analysis revealed a third wave characterized by the Delta VOC, introduced mainly from Turkey and the United States of America, which subsequently spread in the country [48]. The Delta sequences (assigned to 25 sublineages) carried the additional Del156-157 plus the R158G substitution in the S protein and the usual D63G, R203M, and D377Y substitutions in the N protein [48].

In conclusion, this study provides additional information into SARS-CoV-2 epidemiology in Turkey. The patients, with clinical information available, reported specific symptoms of the Delta VOC. Phylogenetic analysis identified nine clusters characterized by viruses isolated in Kahramanmaraş city. No clusters were intermixed with sequences from different regions of Turkey, validating a local transmission of infection. Sanger sequencing detected the usual mutations of the Delta VOC still maintained in the Omicron lineage and sublineage, such as L452R, T478K, D614G, and P681R mutations, resulting in a fitness advantage. Alternatively, ten unusual mutations were carried by new isolates in a restricted time span (one month), suggesting pressure to select specific S and N mutations, such as T696S in the S1/S2 cleavage site, V915I in HR1, and T24I and A359S, able to destabilize the N protein structure, as reported above. Even if Delta disappeared from the global landscape, the same mutation could be selected by new SARS-CoV-2 VOCs. Novel selected mutation during viral replication could impact on the transmissibility, pathogenicity of infection, and performance of molecular or antigenic diagnostic tests, since both the S and N genomic regions are widely used in clinical practice to diagnose SARS-CoV-2 infection. Despite the analysis being performed on samples collected in August 2021, our molecular investigation highlights the need to improve the power of sequencing worldwide and to monitor viral evolution in different geographic areas.

## Figures and Tables

**Table 1:** Patients’ characteristics at SARS-CoV-2 diagnosis.

	Total (%)		Male (%)		Female (%)	
Number of Cases	58	100	27	46.6	31	53.4
Age (Years-Old)						
0–9	1	1.7	1	100.0	0	0.0
10–19	14	24.1	6	42.9	8	57.1
20–29	9	15,5	5	55.6	4	44.4
30–39	14	24.1	7	50.0	7	50.0
40–49	10	17.2	5	50.0	5	50.0
50–59	3	5.2	1	33.3	2	66.7
60–69	5	8.6	1	20.0	4	80.0
≥70	2	3.4	1	50.0	1	50.0
**Clinical manifestations** *						
Aspecific symptoms	2	3.4	0	0.0	2	100.0
Backache	1	1.7	0	0.0	1	100.0
Acute pain + malaise + fatigue	1	1.7	0	0.0	1	100.0
COVID-19 symptoms	34	58.6	18	52.9	16	47.1

* Note: clinical manifestations were not available for 20 patients.

## Data Availability

The newly generated sequences were submitted to the GenBank^®^ database and can be retrieved under accession numbers OQ608097-OQ608154 (spike sequences) and OQ608155-OQ608212 (nucleocapsid sequences).
